# The mixed quantum Rabi model

**DOI:** 10.1038/s41598-019-54756-0

**Published:** 2019-12-04

**Authors:** Liwei Duan, You-Fei Xie, Qing-Hu Chen

**Affiliations:** 1 Department of Physics and Zhejiang Province Key Laboratory of Quantum Technology and Device, Zhejiang University, Hangzhou, 310027 China; 2 Collaborative Innovation Center of Advanced Microstructures, Nanjing University, Nanjing, 210093 China

**Keywords:** Quantum optics, Quantum optics, Quantum simulation

## Abstract

The analytical exact solutions to the mixed quantum Rabi model (QRM) including both one- and two-photon terms are found by using Bogoliubov operators. Transcendental functions in terms of 4 × 4 determinants responsible for the exact solutions are derived. These so-called *G*-functions with pole structures can be reduced to the previous ones in the unmixed QRMs. The zeros of *G*-functions reproduce completely the regular spectra. The exceptional eigenvalues can also be obtained by another transcendental function. From the pole structure, we can derive two energy limits when the two-photon coupling strength tends to the collapse point. All energy levels only collapse to the lower one, which diverges negatively. The level crossings in the unmixed QRMs are relaxed to avoided crossings in the present mixed QRM due to absence of parity symmetry. In the weak two-photon coupling regime, the mixed QRM is equivalent to an one-photon QRM with an effective positive bias, suppressed photon frequency and enhanced one-photon coupling, which may pave a highly efficient and economic way to access the deep-strong one-photon coupling regime.

## Introduction

The quantum Rabi model (QRM) describes the simplest and at the same time most important interaction between a two-level system (or qubit) and a single-mode bosonic cavity which is linear in the quadrature operators^1^. This model is a paradigmatic one in quantum optics for a long time. It has been reactivated in the past decade, due to the progress in many solid-state devices, such as the circuit quantum electrodynamics (QED)^2,3^, trapped ions^4,5^, and quantum dots^6^, where the strong coupling even ultra-strong coupling has been realized. Here we study a natural generalization of the QRM which exhibits both linear and non-linear couplings between the qubit and the cavity, *i.e*. the mixed QRM having both one- and two-photon terms, with Hamiltonian

1 $$H=-\frac{\Delta }{2}{\sigma }_{x}+\omega {a}^{\dagger }a+{\sigma }_{z}({g}_{1}({a}^{\dagger }+a)+{g}_{2}[{({a}^{\dagger })}^{2}+{a}^{2}]),$$

where Δ is the tunneling amplitude of the qubit, *ω* is the frequency of cavity, *σ*_*x*,*z*_ are Pauli matrices describing the two-level system, *a* (${a}^{\dagger }$) is the annihilation (creation) bosonic operator of the cavity mode, and *g*_1_ (*g*_2_) is the linear (nonlinear) qubit-cavity coupling constant.

The nonlinear coupling appears naturally as an effective model for a three-level system when the third (off-resonant) state can be eliminated. The two-photon model has been proposed to apply to certain Rydberg atoms in superconducting microwave cavities^7,8^. Recently, a realistic implementation of the two-photon QRM using trapped ions has been proposed^9^. In the trapped ions, the atom-cavity coupling could be tuned to the collapse regime.

The mixed QRM described by Eq. (1) can also be implemented in the proposal of the circuit QED^10^ if non-zero DC current biases are applied. Using alternative methods, both linear and nonlinear interaction terms can be present in different circuit QED setup by Bertet *et al*.^11,12^. Besides the one-photon process, the two-photon process was also detected in the superconducting qubit and oscillator coupling system^13^. It was shown recently that a general Hamiltonian realized in the microwave driven ions can be used to simulate the QRM with nonlinear coupling^14^ by chosing properly the time dependent phase and in a suitable interaction picture. The combined linear and non-linear couplings can also be attained. More recently, Pedernales *et al*. proposed that a background of a (1 + 1)-dimensional black hole requires a QRM with both one- and two-photon terms that can be implemented in a trapped ion for the quantum simulation of Dirac particles in curved spacetime^15^. So the QRM with both one- and two-photon couplings is not only a generic model in the circuit QED and trapped ions, but also has applications in other realm of physics.

The unmixed QRMs, where either linear or nonlinear coupling is present, have been extensively studied for a few decades (for a review, please refer to refs. ^16–18^). The solution based on the well-defined *G*-function with pole structures was only found for one-photon model by Braak^19^ in the Bargmann representation and two-photon model by Chen *et al*.^20^ using Bogoliubov transformations. These solutions have stimulated extensive research interests in the exact solutions to the unmixed QRMs and their variants with either one-photon^21–26^ or two-photon term^27–34^. In the literature, many analytical approximate but still very accurate results have also been given^35–47^. In some limits of model parameters, the dynamics and quantum criticality have been studied exactly as well^48–50^. In the unmixed QRMs, the parity symmetry is very crucial to get the analytical solution in the closed system. Recently, the role of the parity symmetry has been characterized in the excitation-relaxation dynamics of the system as a function of light-matter coupling in the open systems^51^.

In the mixed QRM with both linear and nonlinear couplings, the parity symmetry is however broken naturally, and the analytical solution thus becomes more difficult^52^, compared to the unmixed models. In this paper, we propose an analytical exact solutions to this mixed QRM. We derive a G-function by the Bogoliubov transformations, which can be reduced to the previous G-functions for both one- and two-photon QRMs if either of the couplings appears. We demonstrate that the derived *G*-function can really yield the regular spectra by checking with the numerics. The exceptional eigenvalues are also given with the help of the non-degeneracy property in this mixed model due to the absence of any symmetry. Two kinds of formulae for the collapse points are derived. The avoided crossings are confirmed. The level collapse in the strong two-photon coupling regime is also discussed. Finally, we study the influence of mixed coupling by constructing an equivalent one-photon QRM with an effective positive bias where the photon frequency is suppressed and the one-photon coupling is enhanced.

## Solutions within the Bogoliubov Operators Approach

In the basis of spin-up and spin-down states, the Hamiltonian (1) can be transformed to the following matrix form in units of *ω* = 1

2 $$H=(\begin{array}{ll}{a}^{\dagger }a+{g}_{1}({a}^{\dagger }+a)+{g}_{2}[{({a}^{\dagger })}^{2}+{a}^{2}] & -\frac{\Delta }{2}\\ -\frac{\Delta }{2} & {a}^{\dagger }a-{g}_{1}({a}^{\dagger }+a)-{g}_{2}[{({a}^{\dagger })}^{2}+{a}^{2}]\end{array}).$$

First, we perform Bogoliubov transformation

3 $$\begin{array}{lllll}A & = & S(r){D}^{\dagger }(w)aD(w){S}^{\dagger }(r) & = & ua+v{a}^{\dagger }+w,\\ {A}^{\dagger } & = & S(r){D}^{\dagger }(w){a}^{\dagger }D(w){S}^{\dagger }(r) & = & u{a}^{\dagger }+va+w,\end{array}$$

to generate a new bosonic operator, where *S*(*r*) is the squeezing operator and *D*(*w*) is the displaced operator

$$S(r)={e}^{\frac{r}{2}({a}^{2}-{a}^{\dagger 2})},\,D(w)={e}^{w({a}^{\dagger }-a)},$$

with $r=arc\,\cosh \,u$. If set

4 $$u=\sqrt{\frac{1+\beta }{2\beta }},v=\sqrt{\frac{1-\beta }{2\beta }},w=\frac{{u}^{2}+{v}^{2}}{u+v}{g}_{1},$$

with $\beta =\sqrt{1-4{g}_{2}^{2}}$, we have a simple quadratic form of one diagonal Hamiltonian matrix element

5 $${H}_{11}=\frac{{A}^{\dagger }A-{v}^{2}-{w}^{2}}{{u}^{2}+{v}^{2}}.$$

The eigenstates of *H*_11_ are the number states |*n*〉_*A*_ which can be written in terms of the Fock states |*n*〉 in original bosonic operator *a* as

6 $${|n\rangle }_{A}=S(r){D}^{\dagger }(w)|n\rangle .$$

Similarly, we can introduce another operator

7 $$\begin{array}{lllll}B & = & {S}^{\dagger }(r){D}^{\dagger }(w^{\prime} )aD(w^{\prime} )S(r) & = & ua-v{a}^{\dagger }+w^{\prime} ,\\ {B}^{\dagger } & = & {S}^{\dagger }(r){D}^{\dagger }(w^{\prime} ){a}^{\dagger }D(w^{\prime} )S(r) & = & u{a}^{\dagger }-va+w^{\prime} ,\end{array}$$

with

$$w^{\prime} =\frac{{u}^{2}+{v}^{2}}{v-u}{g}_{1},$$

which yields a simple quadratic form of the other diagonal Hamiltonian matrix element

8 $${H}_{22}=\frac{{B}^{\dagger }B-{v}^{2}-{w}^{^{\prime} 2}}{{u}^{2}+{v}^{2}}.$$

Note that if *g*_2_ = 0, we have *w* = *g*_1_ and *w*′ = −*g*_1_, which are exactly the same as those in the one-photon QRM^20^. Similarly, the eigenstates of *H*_22_ are the number states |*n*〉_*B*_ which can be written in terms of the Fock states |*n*〉 in original bosonic operator *a* as

9 $${|n\rangle }_{B}={S}^{\dagger }(r){D}^{\dagger }(w^{\prime} )|n\rangle .$$

In terms of the Bogoliubov operator *A*, the Hamiltonian can be written as

10 $$H=(\begin{array}{ll}\frac{{A}^{\dagger }A-{v}^{2}-{w}^{2}}{{u}^{2}+{v}^{2}} & -\frac{\Delta }{2}\\ -\frac{\Delta }{2} & \,{H^{\prime} }_{22}\end{array}),$$

where

11 $${H^{\prime} }_{22}=(({u}^{2}+{v}^{2})+4{g}_{2}uv){A}^{\dagger }A-2uv({({A}^{\dagger })}^{2}+{A}^{2})-2{(u-v)}^{2}w({A}^{\dagger }+A)+{h}_{A},$$

with

$${h}_{A}={v}^{2}+{(u-v)}^{2}{w}^{2}(1-2{g}_{2})+2{g}_{1}(u-v)w+2{g}_{2}uv.$$

In principle, the eigenfunctions of the Hamiltonian can be expanded in terms of the number states of operator *A*

12 $${|\psi \rangle }_{A}=\mathop{\sum }\limits_{n=0}^{\infty }\,\sqrt{n!}(\begin{array}{l}{e}_{n}{|n\rangle }_{A}\\ {f}_{n}{|n\rangle }_{A}\end{array}).$$

Projecting the Schr*ö*dinger equation onto |*n*〉_*A*_ gives

13 $${e}_{n}=\frac{\Delta /2}{\frac{n-{v}^{2}-{w}^{2}}{{u}^{2}+{v}^{2}}-E}{f}_{n},$$

14 $${f}_{n+2}=\frac{-\frac{\Delta }{2}{e}_{n}+[\Omega (n,E)+{h}_{A}]{f}_{n}-2{(u-v)}^{2}w({f}_{n-1}+(n+1){f}_{n+1})-2uv{f}_{n-2}}{2uv(n+1)(n+2)},$$

with

$$\begin{array}{rcl}\Omega (n,E) & = & ({u}^{2}+{v}^{2}+4{g}_{2}uv)n-E\\  & = & \frac{(1+4{g}_{2}^{2})}{\beta }n-E.\end{array}$$

This is actually a five terms recurrence relation for *f*_*n*_. All coefficients *e*_*n*_ and *f*_*n*_ for *n* > 1 are determined in terms of *f*_0_ and *f*_1_ linearly.

In terms of the Bogoliubov operator *B*, the Hamiltonian can be written as

$$H=(\begin{array}{ll}\,{H^{\prime} }_{11} & -\frac{\Delta }{2}\\ -\frac{\Delta }{2} & \frac{{B}^{\dagger }B-{v}^{2}-{w}^{^{\prime} 2}}{{u}^{2}+{v}^{2}}\end{array}),$$

where

$${H^{\prime} }_{11}=(({u}^{2}+{v}^{2})+4{g}_{2}uv){B}^{\dagger }B-2{(u+v)}^{2}w^{\prime} ({B}^{\dagger }+B)+2uv({({B}^{\dagger })}^{2}+{B}^{2})+{h}_{B},$$

with

$${h}_{B}={v}^{2}+{(u+v)}^{2}{w}^{^{\prime} 2}(1+2{g}_{2})-2{g}_{1}(u+v)w^{\prime} +2{g}_{2}uv.$$

We can express the eigenfunctions as

15 $${|\psi \rangle }_{B}=\mathop{\sum }\limits_{n=0}^{\infty }\,\sqrt{n!}(\begin{array}{l}{f^{\prime} }_{n}{|n\rangle }_{B}\\ {e^{\prime} }_{n}{|n\rangle }_{B}\end{array}).$$

Similarly, we can get

16 $${e^{\prime} }_{n}=\frac{\frac{\Delta }{2}}{\frac{n-{v}^{2}-{w}^{^{\prime} 2}}{{u}^{2}+{v}^{2}}-E}{f^{\prime} }_{n},$$

and the similar five terms recurrence relation for ${f^{\prime} }_{n}$. Analogously, all coefficients ${e^{\prime} }_{n}$ and ${f^{\prime} }_{n}$ for *n* > 1 are determined through ${f^{\prime} }_{0}$ and ${f^{\prime} }_{1}$ linearly.

Except for the crossing points in the energy spectra, the eigenstates are nondegenerate. Two wavefunctions in terms of operators *A* and *B* correspond to the same eigenstate. Therefore, they should be proportional with each other by a constant *r*,

17 $$\mathop{\sum }\limits_{n=0}^{\infty }\,\sqrt{n!}(\begin{array}{l}{e}_{n}{|n\rangle }_{A}\\ {f}_{n}{|n\rangle }_{A}\end{array})=r\mathop{\sum }\limits_{n=0}^{\infty }\,\sqrt{n!}(\begin{array}{l}{f^{\prime} }_{n}{|n\rangle }_{B}\\ {e^{\prime} }_{n}{|n\rangle }_{B}\end{array}).$$

We will set *r* = 1, because only ratios among *f*_0_, *f*_1_, $r{f^{\prime} }_{0}$ and $r{f^{\prime} }_{1}$ are relevant. In this case we can absorb *r* into new ${f^{\prime} }_{0}$ and ${f^{\prime} }_{1}$. Then we have

18 $$\mathop{\sum }\limits_{n=0}^{\infty }\,\sqrt{n!}{e}_{n}|n{\rangle }_{A}=\mathop{\sum }\limits_{n=0}^{\infty }\,\sqrt{n!}{f^{\prime} }_{n}|n{\rangle }_{B},$$

19 $$\mathop{\sum }\limits_{n=0}^{\infty }\,\sqrt{n!}{f}_{n}|n{\rangle }_{A}=\mathop{\sum }\limits_{n=0}^{\infty }\,\sqrt{n!}{e^{\prime} }_{n}|n{\rangle }_{B}.$$

In the unmixed QRM, the well-defined *G*-functions can be derived by using the lowest number state |0〉 in the original Fock basis for the one-photon model^19,20^, and two lowest number states |0〉 and |1〉 for the two-photon model^20,30^. Here, we also project Eqs. (18) and (19) onto two original number states |0〉 and |1〉, and then obtain the following 4 equations

20 $${G}^{(0,0)}=\mathop{\sum }\limits_{n=0}^{\infty }\,\sqrt{n!}[{f}_{n}{\langle 0|n\rangle }_{A}-{e^{\prime} }_{n}{\langle 0|n\rangle }_{B}]=0,$$

21 $${G}^{(0,1)}=\mathop{\sum }\limits_{n=0}^{\infty }\,\sqrt{n!}[{f}_{n}{\langle 1|n\rangle }_{A}-{e^{\prime} }_{n}{\langle 1|n\rangle }_{B}]=0,$$

22 $${G}^{(1,0)}=\mathop{\sum }\limits_{n=0}^{\infty }\,\sqrt{n!}[{e}_{n}{\langle 0|n\rangle }_{A}-{f^{\prime} }_{n}{\langle 0|n\rangle }_{B}]=0,$$

23 $${G}^{(1,1)}=\mathop{\sum }\limits_{n=0}^{\infty }\,\sqrt{n!}[{e}_{n}{\langle 1|n\rangle }_{A}-{f^{\prime} }_{n}{\langle 1|n\rangle }_{B}]=0.$$

They form 4 sets of linear homogeneous equations with 4 unknown variables *f*_0_, *f*_1_, ${f^{\prime} }_{0}$, and ${f^{\prime} }_{1}$. Nonzero solutions require the vanishing of the following 4 × 4 determinant

24 $$G(E)=|{G}_{i,j}|=0,$$

where elements *G*_*i*,*j*_*s* are just coefficients before *f*_0_, *f*_1_, ${f^{\prime} }_{0}$, and ${f^{\prime} }_{1}$ in Eqs. (20–23).

Equation (24) is just the *G*-function of the present mixed QRM. Its zeros thus give all regular eigenvalues of the mixed QRM, which in turn give the eigenstates using Eq. (12) or Eq. (15). Note from the coefficients in Eqs. (13) and (14) that this *G*-function is a well-defined transcendental function. Thus analytical exact solutions have been formally found. In the next section, we will employ it to analyze the characteristics of the spectra.

Note that for the one-photon QRM (*g*_2_ = 0), the parity symmetry leads to

$${e^{\prime} }_{n}=\pm {(-1)}^{n}{e}_{n},{f^{\prime} }_{n}=\pm {(-1)}^{n}{f}_{n}.$$

then Eq. (20) becomes

25 $${G}^{(0,0)}(E)=\mathop{\sum }\limits_{n=0}^{\infty }\,\sqrt{n!}[{f}_{n}{\langle 0|n\rangle }_{A}\mp {(-1)}^{n}{e}_{n}{\langle 0|n\rangle }_{B}],$$

which is just the *G*-function of one-photon QRM (*g*_2_ = 0)^19^. Similarly, Eq. (20) (Eq. (21)) can be reduced to the previous ones^20,30^ of the two-photon QRM (*g*_1_ = 0) in the subspace with even (odd) bosonic number.

In various unmixed QRMs, such as one-photon^19,20^, two-photon^30^ or two-mode^53^ QRM, the coefficients of the eigenstates satisfy a three-term recurrence relation which can be achieved by performing the Bogoliubov transformation. All these *G*-functions with explicit pole structures have been summarized in Eq. (27) of ref. ^53^. But in the mixed model, the Hilbert space cannot be separated into invariant subspaces due to the lack of parity symmetry, so the recurrence relation of the coefficients {*f*_*n*_} is of higher order, as seen in Eq. (14). A similar behavior also happens in the Dicke model^54,55^. The possible reason is that the symmetry does not suffice to label each state uniquely, indicating that mixed QRM is non-integrable according to Braak’s criterion for quantum intergrability^19^.

## Exact Spectra

### Regular spectra

**Figure 1:**
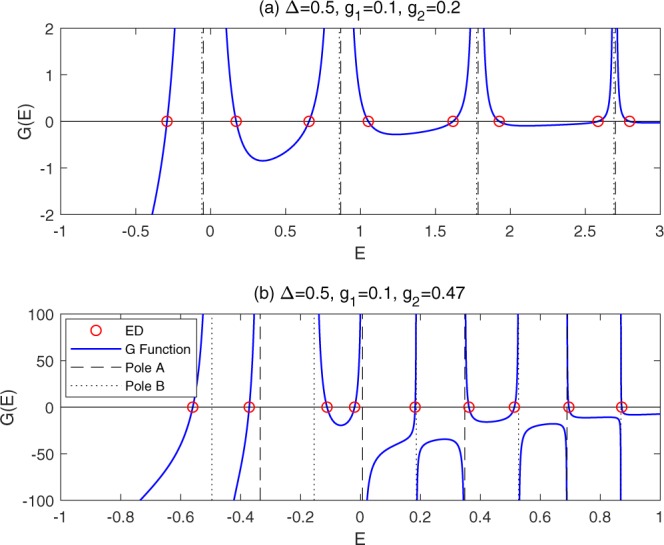
*G*-curves for Δ = 0.5, *g*_1_ = 0.1. (**a**) *g*_2_ = 0.2, (**b**) *g*_2_ = 0.47. The blue solid lines denote the *G*-curves. The dash (dot) lines denote poles associated with *A* (*B*) operator. The red circles denote the results calculated by numerical diagonalization.

To show the validity of the *G*-function (24), we first check with independent numerics. The *G*-curves as a function of *E* for Δ = 0.5, *g*_1_ = 0.1, *g*_2_ = 0.2 and 0.47 are depicted in Fig. 1. We find that the zeros of *G*-function indeed yield the true eigenvalues by comparing with the numerical diagonalization in truncated Hilbert spaces with sufficiently high dimension.

**Figure 2:**
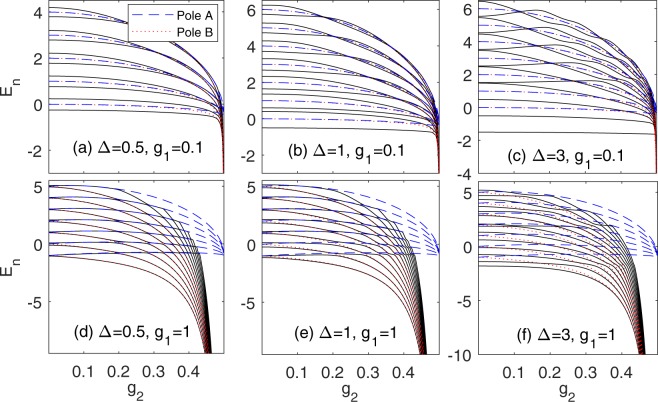
Energy spectra as a function of *g*_2_. The solid black lines denote the energy spectra obtained from the *G*-function. The blue dash (red dot) lines denote poles associated with *A* (*B*) operator.

Then we plot the energy spectra calculated by the *G*-function (24) in Fig. 2. Checking with numerics, our *G*-function reproduces completely all eigenvalues of the present mixed model. When *g*_2_ is close to 1/2, the energy spectra collapse to negative infinity. The parity symmetry in this mixed QRM is lacking, so in principle, the energy degeneracy should be relieved, and level crossings should be absent. However, as shown in Fig. 2(a–c), it seems that some crossings still occur for the small *g*_1_. It will be shown later that these “crossing” can be actually discerned as avoided crossings.

### Pole structure and collapse

From Eqs. (13) and (16), we can find two kinds of poles associated with the *A* and *B* operators respectively, which will lead to the divergency of the recurrence relations,

26 $${E}_{n}^{({\rm{pole}}\_{\rm{A}})}=\beta n-\frac{1-\beta }{2}-\frac{{g}_{1}^{2}}{1+2{g}_{2}},$$

27 $${E}_{n}^{({\rm{pole}}\_{\rm{B}})}=\beta n-\frac{1-\beta }{2}-\frac{{g}_{1}^{2}}{1-2{g}_{2}}.$$

With the same *n*, the difference of two poles is independent of *n*.

$$\Delta {E}^{({\rm{p}})}=\frac{{g}_{1}^{2}}{1-2{g}_{2}}-\frac{{g}_{1}^{2}}{1+2{g}_{2}}={g}_{1}^{2}\frac{4{g}_{2}}{{\beta }^{2}}.$$

In the limit of *g*_2_ → 1/2, *β* → 0, all *E*_*n*_^(pole_A)^ are squeezed into a single finite value $-\frac{1}{2}(1+{g}_{1}^{2})$, while all *E*_*n*_^(pole_B)^ diverge to −∞. It seems that there are two kinds of collapse energies. But actually, all energy levels tend to *B*-poles only, namely −∞, if *g*_2_ → 1/2, as shown in Fig. 2. This spectral characteristics is quite different from the two-photon QRM where the energy spectra collapse to the finite value^9,30,56^. For the mixed QRM, the divergence of the eigenenergies to negative infinity for *g*_2_ → 1/2 suggests some underlying unphysicality, which deserves further studies. The energies of the high excited states cross the pole A curves and then asymptotically converge to the pole B, which lead to exceptional solutions.

### Exceptional solutions

**Figure 3:**
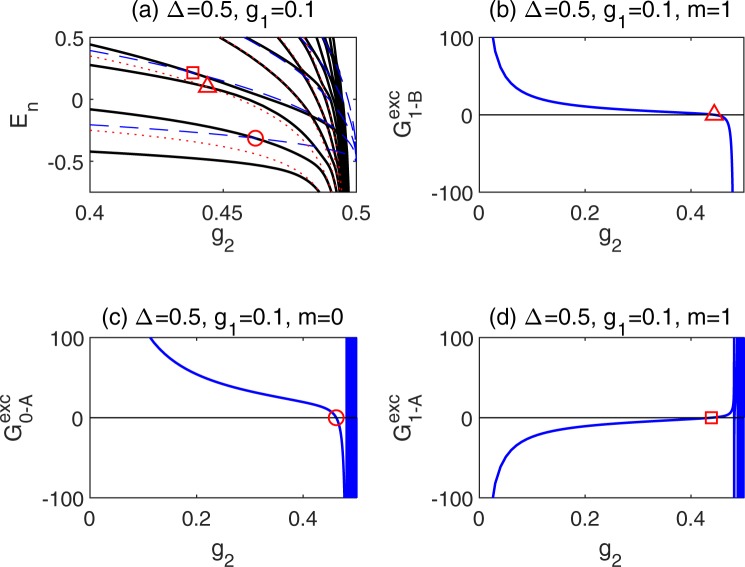
(**a**) Enlarged view of energy spectra in Fig. 2(a). The solid black lines denote the energy spectra obtained from the *G*-function. The blue dash (red dot) lines denote poles associated with *A* (*B*) operator. The triangle denotes the first intersecting point of the energy levels and the 1-st pole line associated with the *B* operator. The circle (square) denotes the intersecting point of the energy level and the 0-th (1-st) pole line associated with the *A* operator. (**b**) The B-type exceptional eigenvalue for *m* = 1. The A-type exceptional eigenvalues for (**c**) *m* = 0 and (**d**) *m* = 1. Δ = 0.5, *g*_1_ = 0.1.

As shown in Fig. 3(a), most energy level curves pass through the pole curves on the way to *g*_2_ = 1/2, which results in so-called exceptional solutions. They can be located in the following way.

At the intersecting point of the energy levels and the *m*-th pole line associated with the *A*-operator (26), the coefficient *f*_*m*_ must vanish so that the pole is lift. Otherwise, the coefficient *e*_*m*_ would diverge due to zero denominator in Eq. (13). In the unmixed QRM, *f*_*m*_ = 0 can uniquely yield the necessary and sufficient condition for the occurrence of the exceptional solution. But here it is not that case, because *f*_*m*_ depends on two initial variable *f*_0_, *f*_1_, and cannot be determined uniquely. The corresponding coefficient *e*_*m*_ should be finite and can be regarded as an unknown variable. In all summations in Eqs. (20)–(23), the *m*-th terms should be treated specially, i.e. let *f*_*m*_ be 0 and *e*_*m*_ be a new variable. By the recurrence relation (14), we can add a new equation for this case

28 $${f}_{m}=\frac{-\frac{\Delta }{2}{e}_{m-2}+[\Omega (m-2,E)+{h}_{A}]{f}_{m-2}-2{(u-v)}^{2}w({f}_{m-3}+(m-1){f}_{m-1})-2uv{f}_{m-4}}{2uvm(m-1)}=0.$$

So for the exceptional solution, we have a set of linear homogeneous equations (Eqs. (20–23) and (28)) with 5 unknown variables *f*_0_, *f*_1_, ${f^{\prime} }_{0}$, ${f^{\prime} }_{1}$ and *e*_*m*_ for *m* ≥ 2. While for 0 ≤ *m* < 2, *f*_*m*_ = 0, we have only 4 unknown variables *f*_1−*m*_, ${f^{\prime} }_{0}$, ${f^{\prime} }_{1}$ and *e*_*m*_, which can be determined by solving another set of linear homogeneous equations (Eqs. (20–23)). Nonzero solution requires the vanishing of the 5 × 5 (4 × 4) determinant whose elements are just coefficients before *f*_0_, *f*_1_, ${f^{\prime} }_{0}$, ${f^{\prime} }_{1}$ and *e*_*m*_ in Eqs. (20–23) and (28) (*f*_1−*m*_, ${f^{\prime} }_{0}$, ${f^{\prime} }_{1}$ and *e*_*m*_ in Eqs. (20–23)) for *m* ≥ 2 (0 ≤ *m* < 2),

29 $${G}_{m-A}^{{\rm{exc}}}(\Delta ,{g}_{1},{g}_{2})=0.$$

We call this function as exceptional *G*-function. Here the energy is not an explicit variable, but determined by Eq. (26). The *m*-th exceptional solution associated with the *B* operator can be detected in the same way by zero of an exceptional *G*-function

30 $${G}_{m-B}^{{\rm{exc}}}(\Delta ,{g}_{1},{g}_{2})=0.$$

With the help of the exceptional *G*-function, we can determine the intersecting points of the energy levels and the pole curves as shown in Fig. 3(a) for Δ = 0.5, *g*_1_ = 0.1. The *G*^exc^ curves associated with *A*-pole as a function of *g*_2_ for *m* = 0 and 1 are shown in Fig. 3(c,d) respectively, and the *G*^exc^ curve associated with *B*-pole for *m* = 1 is shown in Fig. 3(b). The detected exceptional solutions in the *G*^exc^ curves are marked by the same symbols as those in the enlarged spectra graph. One can find many zeros for *G*^exc^ curve associated with *A*-pole in Fig. 3(c,d), which are corresponding to intersecting points of energy levels and the *m*-th *A*-pole curves as displayed in Fig. 3(a). For *G*^exc^ curve associated with *B*-pole, there is only one zero for *m* = 1 as exhibited in Fig. 3(b), also consistent with the single intersecting point shown in Fig. 3(a). No exceptional solution exists even for *B*-pole with *m* = 0. All *g*_2_ obtained from *G*^exc^ = 0 can find their corresponding intersecting points in the energy spectra exactly.

Now we can judge whether it is the true level crossing or avoided crossing in the Fig. 2(a–c). Around this regime, we have not found any exceptional solutions, indicating that the energy level cannot intersect with the pole curves. So although two energy levels are very close but blocked off by two pole curves with difference Δ*E*^(p)^ ∝ *g*_1_^2^, they neither collide nor cross with each other. It is actually avoided crossing. For small *g*_1_, Δ*E*^(p)^ is very small, so it looks like a “level crossing” as depicted in Fig. 2(a–c). For large *g*_1_, the avoided crossing is quite clear, as shown in Fig. 2(d–f). Actually, these avoided crossings are just remnants of the traces of the doubly degeneracy in the unmixed model, which is relieved in the mixed model.

### Effect of the mixed couplings

In the mixed QRM, if we combine Eqs. (5) and (8), the Hamiltonian (Eq. (2)) can be written as

31 $$H=(\begin{array}{ll}\frac{{A}^{\dagger }A-{v}^{2}-{w}^{2}}{{u}^{2}+{v}^{2}} & -\frac{\Delta }{2}\\ -\frac{\Delta }{2} & \,\frac{{B}^{\dagger }B-{v}^{2}-{w}^{^{\prime} 2}}{{u}^{2}+{v}^{2}}\end{array}),$$

which can be reorganized and separated into three terms:

32 $$H={H}_{0}+\frac{{\varepsilon }^{({\rm{eff}})}}{2}{\sigma }_{z}-\frac{1-\beta }{2}{\bf{I}},$$

where ***I*** is a unit matrix, and

33 $${H}_{0}=(\begin{array}{ll}\beta {A}^{\dagger }A-\frac{{g}_{1}^{2}}{{\beta }^{2}} & -\frac{\Delta }{2}\\ -\frac{\Delta }{2} & \,\beta {B}^{\dagger }B-\frac{{g}_{1}^{2}}{{\beta }^{2}}\end{array}),$$

34 $${\varepsilon }^{({\rm{eff}})}=\frac{4{g}_{2}}{1-4{g}_{2}^{2}}{g}_{1}^{2}.$$

An effective bias *ε*^(eff)^ appears naturally, as well as a total energy shift (1 − *β*)/2. Recalling the one-photon QRM^20^,

35 $${H}_{1{\rm{P}}}=-\frac{\Delta }{2}{\sigma }_{x}+\omega {a}^{\dagger }a+{g}_{1}{\sigma }_{z}({a}^{\dagger }+a),$$

this Hamiltonian can be expressed with a new set of bosonic operators $P={D}^{\dagger }({g}_{1})aD({g}_{1})=a+{g}_{1}$ and $Q=D({g}_{1})a{D}^{\dagger }({g}_{1})=a-{g}_{1}$ as

36 $${H}_{1{\rm{P}}}=(\begin{array}{ll}\omega {P}^{\dagger }P-\frac{{g}_{1}^{2}}{\omega } & -\frac{\Delta }{2}\\ -\frac{\Delta }{2} & \,\omega {Q}^{\dagger }Q-\frac{{g}_{1}^{2}}{\omega }\end{array}).$$

Comparing Eqs. (33) and (36), we can introduce an effective photon frequency *ω*^(eff)^ and an effective one-photon coupling strength ${g}_{1}^{(\text{eff})}$,

$${\omega }^{({\rm{eff}})}=\beta ,$$

$${g}_{1}^{({\rm{eff}})}=\frac{{g}_{1}}{\sqrt{\beta }},$$

and rewrite *H*_0_ as

$${H}_{0}=-\frac{\Delta }{2}{\sigma }_{x}+{\omega }^{({\rm{eff}})}{a}^{\dagger }a+{g}_{1}^{({\rm{eff}})}{\sigma }_{z}({a}^{\dagger }+a).$$

Therefore, we construct an effective one-photon QRM to describe the mixed one, which provides a more intuitional description of the influence of the mixed coupling,

37 $$\begin{array}{rcl}{H}^{({\rm{eff}})} & = & \frac{{\varepsilon }^{({\rm{eff}})}}{2}{\sigma }_{z}-\frac{\Delta }{2}{\sigma }_{x}-\frac{1-\beta }{2}\\  &  & +{\omega }^{({\rm{eff}})}{a}^{\dagger }a+{g}_{1}^{({\rm{eff}})}{\sigma }_{z}({a}^{\dagger }+a).\end{array}$$

**Figure 4:**
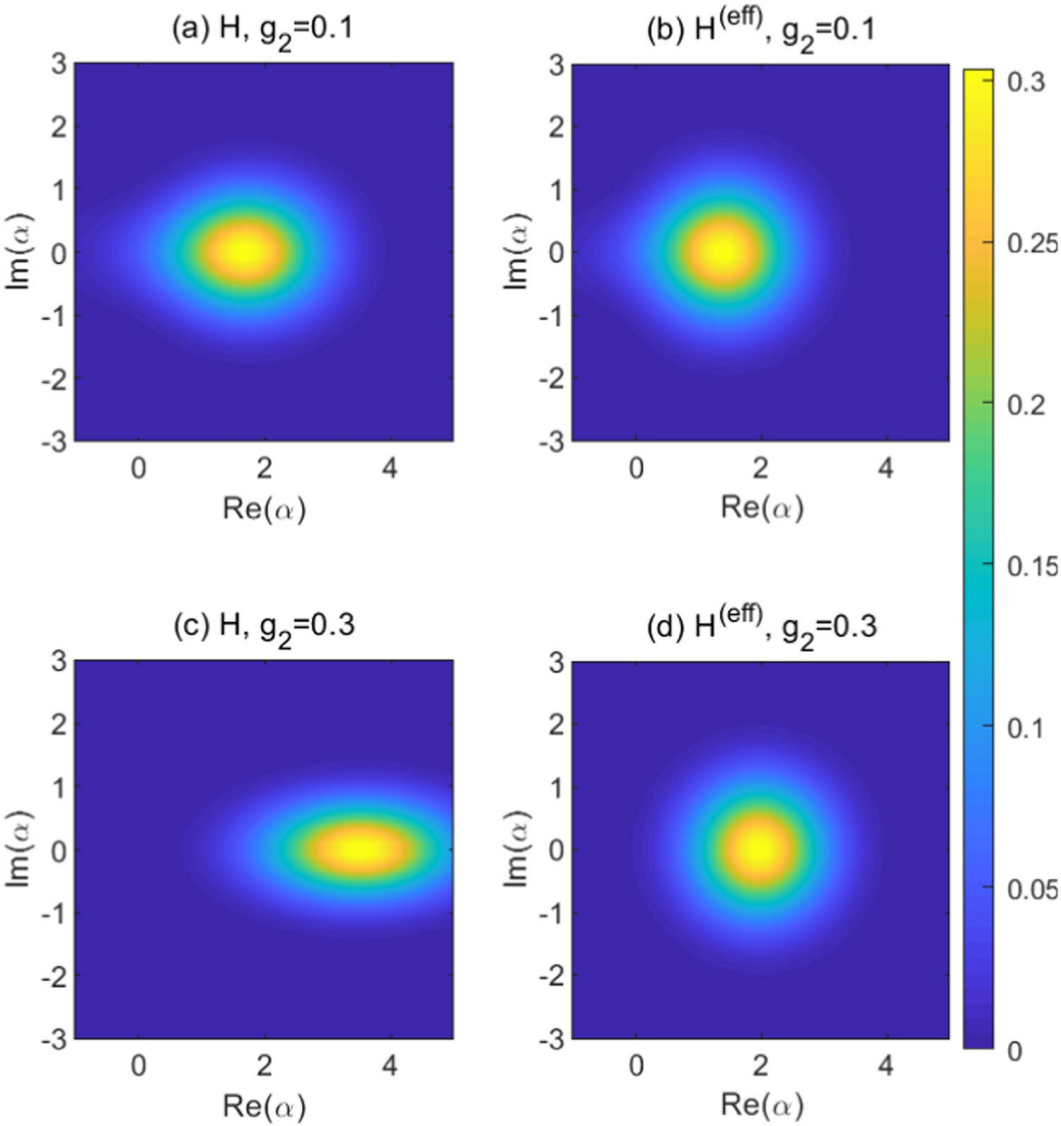
The Wigner function of the ground state at Δ = 1, *g*_1_ = 1, with (**a**,**b**) *g*_2_ = 0.1 and (**c**,**d**) *g*_2_ = 0.3. Left column is calculated from the original Hamiltonian *H*, and the right column is calculated from the effective Hamiltonian *H*^(eff)^.

**Figure 5:**
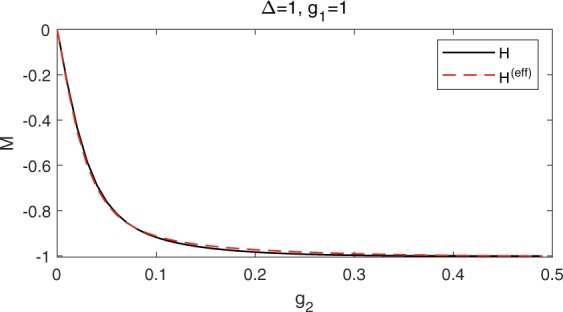
The ground-state magnetization *M* as a function of *g*_2_ for Δ = 1, *g*_1_ = 1. Results for the full mixed model (1) are denoted by black solid lines, agreeing well with those by effective Hamiltonian (37) denoted by red dashed lines.

Comparing *P* (*Q*) with *A* (*B*), the mainly difference is the lack of squeezing operator *S*(*r*). The two-photon interaction leads to the squeezed field state^10,47^, which can be well captured by the squeezing operator^20,30,34^. Therefore, the squeezing operator is introduced explicitly to deal with the mixed QRM, but it is not necessary for the one-photon model. The definitions of *P* (*Q*) in Eq. (33) is equivalent with that of *A* (*B*) in Eq. (36) only if *g*_2_ = 0, so it is hard to use the effective Hamiltonian to deal with the strong two-photon coupling regime, especially the bosonic part due to the intense squeezing effect. We calculate the Wigner function *W*(*α*,α^*^) of the ground state^57,58^ in Fig. 4, which describes the probability distribution of the bosonic field in the phase space. When *g*_2_ = 0.1, the differences of the Wigner functions calculated from *H* and *H*^(eff)^ are negligible. However, It is shown in Fig. 4(c) that the squeezing effect becomes apparent for *H* when *g*_2_ = 0.3. The effective Hamiltonian can hardly describe the squeezing effect as demonstrated in Fig. 4(d). Nevertheless, it shed light on the analysis of strong coupling case, especially the properties of the two-level system. The ground-state magnetization *M* = 〈*ψ*_*GS*_|*σ*_*z*_|*ψ*_*GS*_〉 calculated from *H*^(eff)^ is in good agreement with that calculated from *H* even in strong two-photon coupling regime, as shown in Fig. 5. When *g*_2_ tends to 1/2, the effective bias *ε*^(eff)^ will tend to infinity, and the two-level system will prefer to stay in the lower level as indicated by the ground-state magnetization *M* → −1 in Fig. 5. Therefore, the energy contributed by *ε*^(eff)^*σ*_*z*_/2 would be negative infinite, which is one of the reason for the negative divergence of the eigenenergies in the mixed QRM in the limit of *g*_2_ → 1/2, as observed in Fig. 2. What is more, with the increase of *g*_2_, the effective photon frequency *ω*^(eff)^ will decrease while the effective coupling ${g}_{1}^{(\text{eff})}$ will increase, which might provide a novel and economic way to reach deep-strong one-photon coupling regime.

**Figure 6:**
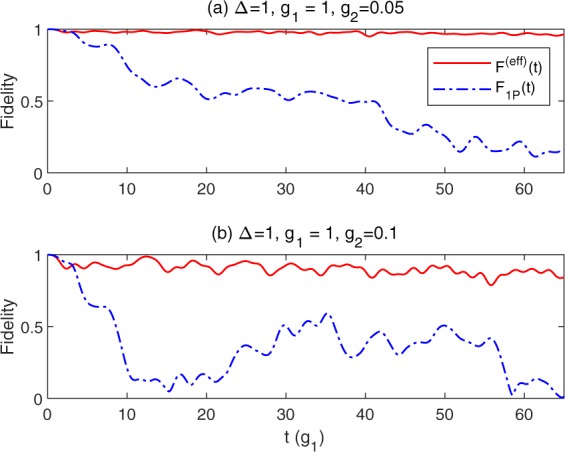
The fidelity of *H*^(eff)^ (red sold line) and *H*_1P_ (blue dash-dot line) as a function of *t* for Δ = 1, *g*_1_ = 1, (**a**) *g*_2_ = 0.05 and (**b**) *g*_2_ = 0.1.

To further demonstrate the accuracy of the effective Hamiltonian, we calculate the dynamics of fidelity as shown in Fig. 6. The fidelity is defined as the overlap of the wavefunctions |*ψ*^(eff)^(*t*)〉 obtained from *H*^(eff)^ (Eq. (37)) and |*ψ*(*t*)〉 obtained from *H* (Eq. (1)), namely *F*^(eff)^(*t*) = |〈*ψ*^(eff)^(*t*)|*ψ*(*t*)〉|, which can be used to judge how accurately the state of the effective Hamiltonian reproduces that of the original Hamiltonian. The initial states are |↑〉|0〉 for both of them. The fidelity of the unbiased one-photon QRM, namely *H*_1P_ (Eq. (35)), is also presented for comparison, *i. e. F*_1*P*_(*t*) = |〈*ψ*_1*P*_(*t*)|*ψ*(*t*)〉|. When *g*_2_ is as small as 0.05, the corresponding effective bias reaches *ε*^(eff)^ ≃ 0.202. This effective bias is large enough to play a significant role in the evolution of fidelity, as clearly seen in the Fig. 6(a). The fidelity of *H*^(eff)^ tends to one, which is a strong evidence of the equivalence between |*ψ*^(eff)^(*t*)〉 and |*ψ*(*t*)〉. The fidelity of *H*_1P_ is much smaller, indicating that it deviates from the original Hamiltonian significantly. When we further increase *g*_2_, the effective Hamiltonian still gives considerably good results while the deviation of *H*_1P_ becomes more obvious. The fidelity of *H*^(eff)^ drops slightly in the long time, which is mainly due to the error accumulation. The results of fidelity confirm the limitation of the effective Hamiltonian in dealing with the strong two-photon coupling and long-time limits.

**Figure 7:**
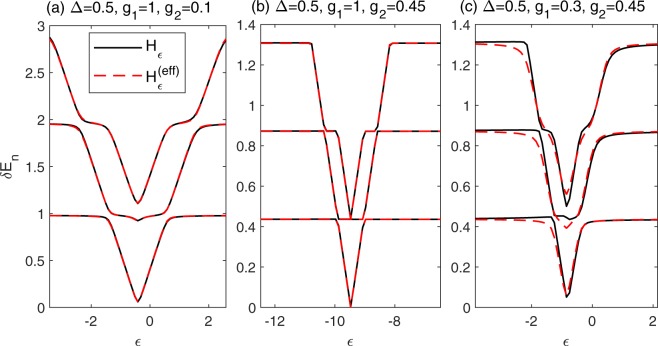
The energy difference *δE*_*n*_ as a function of *ε* for (**a**) Δ = 0.5, *g*_1_ = 1, *g*_2_ = 0.1, (**b**) Δ = 0.5, *g*_1_ = 1, *g*_2_ = 0.45, and (**c**) Δ = 0.5, *g*_1_ = 0.3, *g*_2_ = 0.45. Results calculated from *H*_*ε*_, and *H*_*ε*_^(eff)^ are marked with black solid and red dash lines respectively.

The mixed QRM can be realized in the experiment by coupling the flux qubit to the plasma mode of its DC-SQUID detector^11^. We expect that the effective Hamiltonian can be employed to explain the experimental results. One of the most widely measured quantity in experiments is the transmission spectrum^3,59,60^. The transmission spectrum, *i.e. δE*_*n*_ = *E*_*n*_ − *E*_0_, from both original full model (1) and the effective model (37) are shown in Fig. 7. We introduce an additional bias *εσ*_*z*_/2 which is originated from the an externally applied magnetic flux in circuit QED system, and Eqs. (1) and (37) become

38 $$\begin{array}{rcl}{H}_{\varepsilon } & = & \frac{\varepsilon }{2}{\sigma }_{z}-\frac{\Delta }{2}{\sigma }_{x}+{a}^{\dagger }a\\  &  & +{\sigma }_{z}({g}_{1}({a}^{\dagger }+a)+{g}_{2}[{({a}^{\dagger })}^{2}+{a}^{2}]),\end{array}$$

39 $$\begin{array}{rcl}{H}_{\varepsilon }^{({\rm{eff}})} & = & \frac{\varepsilon +{\varepsilon }^{({\rm{eff}})}}{2}{\sigma }_{z}-\frac{\Delta }{2}{\sigma }_{x}\\  &  & +{\omega }^{({\rm{eff}})}{a}^{\dagger }a+{g}_{1}^{({\rm{eff}})}{\sigma }_{z}({a}^{\dagger }+a).\end{array}$$

Therefore, the total bias in the effective Hamiltonian is (*ε*^(eff)^ + *ε*). It is obvious from Fig. 7(a) that the effective Hamiltonian can capture the effects of two-photon coupling in the weak coupling regime very well. Even for strong two-photon coupling *g*_2_ = 0.45, the effective Hamiltonian (39) still provides quite accurate energy structure and almost captures all features of the mixed QRM, as shown in Fig. 7(b,c). With the decrease of *g*_1_, the deviation appears gradually because the two-photon interaction becomes dominated. One should also note that the energy differences of the effective Hamiltonian (Eq. (39)) is symmetry about *ε* = −*ε*^(eff)^, which is different from that of the original one (Eq. (38)), as shown in Fig. 7(c). For the effective Hamiltonian with an additional bias, we can easy confirm that only the absolute value of (*ε* + *ε*^(eff)^) affects the eigenenergies, as well as the energy differences. For the mixed QRM, this symmetry is broken due to the two-photon interaction term. Therefore, the asymmetry in the transmission spectrum can be regarded as a signature of the mixed one- and two-photon couplings. Far from the symmetry point *ε* = −*ε*^(eff)^, the energy difference tends to be a multiple of the photon frequency. The energy difference decreases with the increase of *g*_2_, which can be explained by the suppressed effective photon frequency *ω*^(eff)^.

**Figure 8:**
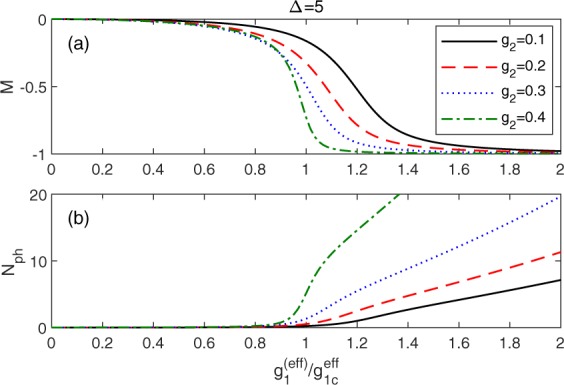
(**a**) The magnetization *M* and (**b**) photon number *N*_ph_ calculated from *H* as a function of ${g}_{1}^{(\text{eff})}$ for Δ = 5, *g*_2_ = 0.1 (black solid), 0.2 (red dash), 0.3 (blue dot) and 0.4 (green dash-dot lines).

Recently, the quantum phase transition of one-photon QRM in Δ/*ω* → ∞ has drawn much attention^48,49^. The magnetization *M* = 〈*σ*_*z*_〉 serves as an order parameter which changes from zero in the normal phase to nonzero in the superradiant phase when *g*_1_ crosses the critical point ${g}_{1c}=\sqrt{\Delta \omega }/2$. Above *g*_1*c*_, photons are extremely activated as well. The ground-state magnetization *M* and the photon number ${N}_{{\rm{ph}}}=\langle {a}^{\dagger }a\rangle$ calculated from *H* (Eq. (1)) as a function of the scaled coupling ${g}_{1}^{(\mathrm{eff})}/{g}_{1c}^{(\mathrm{eff})}$ for large values of Δ are shown in Fig. 8, where ${g}_{1c}^{({\rm{eff}})}=\sqrt{\Delta {\omega }^{({\rm{eff}})}}/2$. A negative *M* emerges in the mixed QRM due to the positive effective bias. Clearly, the photon number *N*_ph_ increases with the increase of *g*_2_, which also indicates that the qubit-cavity interactions are enhanced and more photons are excited. For different two-photon coupling in the mixed QRM, the photons are considerably enhanced at almost the same scaled coupling around 1. Whether it is a signature of the quantum phase transitions in the mixed QRM as observed in ref. ^50,61^. deserves further study.

## Summary

In this paper, by using Bogoliubov operators, we exactly solve the mixed QRM with both one- and two-photon terms analytically. The *G*-functions with the pole structures are derived, which reproduce completely the regular spectra. They can also be reduced to the unmixed ones. It is found that there are two sets of poles associated with two Bogoliubov operators. Two types of exceptional eigenvalues are then derived, which cannot be obtained solely by requiring that the corresponding coefficients vanish like in the unmixed models. When the two-photon coupling strength *g*_2_ is close to 1/2, two collapse energies are derived. One is finite, while the other diverges negatively. All energy levels collapse to the lower one, therefore diverge also, in sharp contrast to the unmixed two-photon model. The level degeneracy in the unmixed model is relieved due to the absence of parity symmetry. The avoided crossings are strictly discerned from the very close levels in the mixed model by the absence of exceptional eigenvalues around the “crossings”.

We construct an effective one-photon Hamiltonian to describe the the mixed QRM, which is valid in weak two-photon coupling regime. The mixed QRM is equivalent to a single-photon one with an effective positive bias, suppressed photon frequency and enhanced one-photon coupling. This feature in the mixed system is very helpful to the recent circuit QED experiments where the intense competition to increase one-photon coupling is performed in many groups^2,3,59,60^. We suggest that the simultaneous presence of both one- and two-photon couplings would cooperate to provide richer physics.
